# Academy of Stomatology

**Published:** 1900-01

**Authors:** 

**Affiliations:** Academy of Stomatology


### ACADEMY OF STOMATOLOGY.

THE regular monthly meeting of the Academy of Stomatology was held at the rooms of the Academy, 1731 Chestnut Street, on the evening of October 24, the Vice-President, Dr. J. T. Lippincott, in the chair.

A general discussion upon the subject of "Cavity Preparation upon Mathematical Lines" was opened by Professor S. H. Guilford.

*Professor Guilford.*—The president of the society called me up over the telephone last week and asked me to prepare something for this meeting. I declined, because I felt that my hands were full. However, he seemed to feel that the first meeting of the year should not be devoted entirely to sociability, as it would be a bad beginning. So I finally consented to speak upon this subject, which was of his selection. I did not have time to prepare a paper, nor to prepare myself adequately to speak upon the subject suggested. I have simply reviewed it in the time I had, and selected some models and charts I happened to have at the college to illustrate my remarks.

Cavity preparation upon mathematical lines is a subject that has interested our scientific men during the past few years, but has never received much attention at the hands of the profession in general.

When Dr. Black was preparing his work on the forms of teeth, he felt interested in having some accurate measurements in order to prosecute the work. For that purpose he used an instrument, the cavity micrometer, and calipers for making these measurements. A year and a half ago, while preparing a paper on instruments, he used a millimetre gauge. It had occurred to Dr. Wedelstaedt, of St. Paul, to measure cavities by this instrument, and he seemed to think an advantage could be gained by measuring in a very exact way. He said, first of all, that the dental profession was not scientific enough nor exact enough; that we were in the habit of talking loosely and without conveying exact ideas; that all we talked about was large cavities, medium-sized cavities, and small-sized cavities; that those terms did not express anything definite, and the only way to get at the matter was to make measurements and express what was meant in millimetres. He also said that if anybody could point out to him what was meant by a medium-sized cavity he would be grateful to him. So he took up the matter and had an instrument made that would measure the depth of a cavity, and he made measurements with this instrument, and afterwards read a paper before a dental society in which he detailed his work, presented a tabulation of his measurements, and argued the advantage of measuring cavities carefully.

He claimed that dentists were unable to state the exact size of a filling to be put in a tooth without a correct measurement, or to know at once what amount of gold it would take to fill a cavity. Consequently, he declared, it would be more scientific if we had some data to go by. He spent a great deal of time over the matter, but, judging by the discussion that followed the reading of the paper, it failed to make an impression as to its practical value.

With regard to the crown of the tooth, much was done by Dr. Black, but he did not carry his work beyond reasonable limits. He thought that in making a section of a tooth it would be very well to be able to know the thickness at certain points, especially with relation to the pulp. All these things were very well to know, but I do not think he convinced any one that there was any practical value in it, although he spent a good deal of time upon it. I simply mention this matter in order to get the opinions of those who are present. For myself I would say that dental cavities vary so much in their outlines that measurements would be of very little value.

In preparing an occlusal cavity we do not care so much for the dimensions as we do for its proper cleansing, extension, and shaping. In many cases the decay has progressed to different degrees of depth, so in measuring we might measure to the deepest point. In such a case it would not do much good if the cavity were mapped out upon the record. Where we have a flat floor we may make a measurement, but in seeking to get a flat surface we may be inclined to cut away a great deal more of the tooth than we would be justified in doing.

Dr. Wedelstaedt mentioned the matter in connection with the identification of dead bodies, and that after a cavity is prepared a measurement from the cavity floor to the gum line should be taken, and then the cavity filled up with whatever material it is decided to use, when a measurement from the occlusal surface to the gum line is to be taken and these measurements recorded. He said that in this way one would have a record of everything done for each individual which would prove valuable in court.

He did not make any explanation of how a record of one dimension of a cavity would be of any particular use. The only dimensions one would really have would be those of the outside. He spoke of taking a vertical and transverse measurement, and seemed to be satisfied with them, though they amount to nothing, as they do not give any idea of the cavity. An occlusal cavity may have these two dimensions and yet be oblong in form or oval or perfectly round. If a cavity is to be correctly described one must have a great many dimensions.

Dr. Wedelstaedt went so far as to say that, by measuring a great number of cavities and crowns of teeth in which they occurred, he had been able to fix a definite rule for the size of the cavity. He said that by measuring the length of the enamel on the proximal surface and the greatest width of the tooth, and by taking two-thirds of each measurement, he could get the exact size of the cavity required for a filling that would obviate recurrence of decay. In other words, if the length of the proximal enamel was 9.9 millimetres and the width 7.2, that the cavity ought to be of a size two-thirds of each,—that is, 6.6 long and 4.8 wide.

This brings up another matter that has interested the Western men for some time past, and that is the point which was brought out by Dr. Black in some of his articles in the *Dental Cosmos,* in which he wrote of "extension for prevention." These words had a very smooth sound, and conveyed a definite idea. The idea was that during preparation a cavity should be so extended as to include not only all of the injured surface and the contact point, but to lines which would cause the margins of fillings to be beyond contact from the adjoining teeth and likewise be cleansed by the friction of food during mastication, and that the filling when completed should be as nearly perfect as possible to prevent recurrence of decay.

In speaking of proximal cavities he mentions that on these surfaces of teeth we have an oval contact point, and just above that is the point of the commencement of caries. Now, in preparing a cavity of that kind he felt that we should not only cut away the injured portion actually decayed, and include the contact point, but extend the excavation freely, buccally, lingually, and cervically, making a wide, flat base cervically. He claimed that if the cavity be prepared in the ordinary way,—rounded at the cervical margin,—an area of tooth structure in proximity to the linguo-cervical and labio-cervical angles of the cavity would remain, which, while not showing that it had been affected by caries, would invariably decay in the course of time.

I believe cavities should be extended to include this area.

He advocated extending the cervical wall beneath the gum, as such extension was seldom followed by a recurrence of decay.

He also urged the extension of occlusal cavities to include all the fissures, as otherwise there would be no security against subsequent decay.

When Dr. Wedelstaedt read his paper he was asked whether this plan met with his approval, and he said it did. He evidently believed in it, because he argued on that line all the way through. Whenever the cavity extends nearly to the gum-line there will be no difficulty in cutting beyond it. The question is, first, whether we are justified in doing the cutting, and, secondly, whether the patient will submit to it. Again, the patient may think that, instead of practising extension for prevention, we are practising extension for remuneration, and be dissatisfied.

A few years ago, when I was in Chicago, the question of filling displacements came up, and Dr. Black said that fillings placed in cavities that are rounded at the cervical margin are liable to be displaced, that stress upon the filling would be liable to cause it to turn in its socket or to displace it, and that in order to avoid such an accident the cervical margin should be made flat.

The explanation given as to how this could occur was not satisfactory. I have never had any trouble with displacements in either form of preparation; and if it has not been my experience, I do not see why it should be that of others. In proximal cavities, when necessity for access demands it, or fracture of the occlusal wall is threatened as a future possibility, the cavity should, as a rule, be extended so as to include the occlusal sulcus. This extension gives ample anchorage to the filling, and I have never had such a filling displaced. I cannot bring myself to believe that it is right, even if the patient submit to it, to convert a small cavity into a large one in order to make it more secure. We fill cavities of all sizes, and they last a long time. If in the course of time decay recur, it is not a difficult matter to cut out the injured portion and add to the filling. If made large at first, and in the course of time the filling has to be renewed, it is a very difficult matter to make another filling larger than before.

As to lateral extension along the cervical margin, I see no necessity for it, and cannot bring myself to do it.

Now, gentlemen, I have opened this subject, and I hope it will be thoroughly discussed.

*The Vice-President.*—Dr. Guilford has given us an interesting talk on this subject, and we will be glad to hear from any one else.

*Dr. Darby.*—This subject has been an interesting one. I presume the articles Dr. Guilford refers to are those of Dr. Wedelstaedt, published in the *Dental Cosmos* for December, 1897. The articles in themselves are exceedingly ingenious, very scientific, to the point, and interesting. Dr. Guilford has given us a very accurate description of these papers, and has treated the subject in a very conservative way. There are two sides to every question, and there are two sides to this. I suppose that if we were to analyze Dr. Wedelstaedt's mind we should find him to have a scientific bent, and that he had treated the subject from this stand-point. As an extremist he is correct. We all know that teeth do quite often fail because we do not cut them enough. I have heard it said that we ought not to cut them too much. And I have heard it said that we cut too little. In the later years of life I know we cut very freely. I do not cut, nor does the average man who saves teeth, cut so deeply as Dr. Wedelstaedt advocates. I want to be fully justified by reason in cutting. I occasionally see teeth that were filled forty years ago by old Philadelphia practitioners still doing good service. The cavities were not much larger than two pin-heads placed side by side, but the fillings are still as good as when they were put in. I suppose that if the patients had been in the hands of Dr. Wedelstaedt he would have cut considerably deeper, according to his rule, and to certain lines. I do not think we are entitled to do that always; yet I have no doubt Dr. Wedelstaedt is correct in so far as he advocates cutting freely.

In proximal cavities I do not think it necessary to cut that we may get a flat surface across the cervical border. It is true that if we cut away there we preclude the recurrence of decay at points along the lingual and the buccal margins of the cavity, where we find very frequently that we have fillings to repair. It is to avoid such vulnerable points as these that Dr. Wedelstaedt advocates his practice. To my mind there is a medium ground to be taken in the preparation of cavities. I do not think we are justified in cutting a tooth to pieces for fear of possible decay. It is better to take the chances and advocate care upon the part of the patients.

I place no confidence in the millimetre measurement. We all have good practical common sense, and use that common sense in making the cavity and packing the gold. We have saved teeth for half a century or more. It is not necessary to go to so much trouble to ascertain what size a cavity should be, and I think that perhaps the thing is impracticable.

Dr. Webb advocated the use of the micrometer, and said that we should extend our cavities freely to save the teeth. We know of no man who cut as freely as Dr. Webb. Dr. Gardiner ought to say something to us that will be valuable.

*Dr. Gardiner.*—I never could bring myself to cut teeth in the manner advocated by Dr. Black and Dr. Wedelstaedt. I believe in many cases in extending the cavity to prevent decay, but I should be governed by the character of the teeth and the condition of the cavity as found.

I do not feel justified in making a large cavity from a small pin-head cavity. There is no necessity of doing it, and no justice in charging for such service when not required.

In proximal cavities of molars reaching a moderate size, I think it is better usually to extend enough to free the margin from contact, but it is not necessary to carry these cavities beneath the gum. In large-sized cavities I like to bevel all the walls outwardly, so as to protect the margins without weakening the teeth. If your cavities are extended as Dr. Black and Dr. Wedelstaedt advocate, and steps made, teeth are weakened to a very marked degree, especially serious in bicuspids. If much decay exists, then I would cut freely without hesitation, but I would not cut away a large portion of the tooth-substance unless I thought it absolutely necessary. In cavities of medium size I would not consider it advisable to cut. I can say, I think without fear of contradiction, that all radical methods have been failures, particularly in the cutting of teeth. I believe a conservative treatment is always best.

*Dr. A. H. Thompson.*—I can add very little to what has been said concerning the measurement of cavities. At the time Dr. Wedelstaedt prepared his paper I felt a great deal of interest in the subject, but it did not seem to me to have any practical value.

Extension for prevention was fully brought out by Dr. Black, but may be carried too far. It is true that a collection will form upon the metal and not upon the teeth, and so will not lead to any harm. The shaping of the proximal surface in this manner is a great labor. The only point I can make is that it is valuable that the contact point shall be reduced to be as small as possible to make it more readily cleansed, which is essential for the prevention of recurrence of decay.

*Dr. W. H. Trueman.*—I have read the article by Dr. Wedelstaedt to which reference has been made, and I feel that we are not justified in extending our cavities according to measurement. The time may come when we can do it, but until then a discriminating judgment guided by experience will be a far better guide than any other.

The paper referred to, belonging to a series given us during the last few years, seems to be characterized by a mass of impossibilities presented by the writer. He has learned, no doubt, to feel the importance of his suggestions.

Dr. Black has stated that there is nothing at all in the strength of the teeth as we understand it; that they are all equally strong so far as structure is concerned, and able to receive gold fillings.

Now, some of us do not believe that, and to me it seems that while preparing cavities we fail to take into account the capacity of teeth to resist cutting and hammering instruments.

Another point which has been touched upon in the discussion is the malleability of gold. When a gold filling is subjected to the impact of mastication for a time, it will undoubtedly change its shape, so that the fillings in a good many teeth will come loose. Not because they are improperly shaped, not because the tooth has been improperly filled, but because of the impact brought to bear upon the gold. In time it is forced from position by reason of a change in its shape.

I remember some years ago seeing a workman operate upon a piece of metal with a hammer. He sat hour after hour pounding with a heavy hammer a mass of iron six inches in diameter, expecting in a week to force it to fit perfectly the hole in which it only fitted loosely. He had been at it several days. I marvelled at the possibility. I saw it afterwards, and it had swelled out and filled the hole perfectly.

I think in our filling we should remember this, and that it is wise to keep the cavities as small as possible, and utilize extension for prevention only up to a certain point. I have observed that the limit is very soon reached. If we carry it out to the utmost limit, why not put a gold crown on the tooth and have no more difficulty?

*Dr. Guilford.*—I simply desire to say that my whole object was to bring out discussion. I have succeeded, and I am satisfied.

Adjourned.

Otto E. Inglis,
*Editor Academy of Stomatology.*

